# Draft Genome Sequence of *Lachancea lanzarotensis* CBS 12615^T^*, *an Ascomycetous Yeast Isolated from Grapes

**DOI:** 10.1128/genomeA.00292-15

**Published:** 2015-04-16

**Authors:** Véronique Sarilar, Hugo Devillers, Kelle C. Freel, Joseph Schacherer, Cécile Neuvéglise

**Affiliations:** a INRA, UMR1319 Micalis, Jouy-en-Josas, France; b AgroParisTech, UMR1319 Micalis, Jouy-en-Josas, France; c CNRS, Department of Genetics, Genomics and Microbiology, University of Strasbourg, UMR7156, Strasbourg, France

## Abstract

We report the genome sequencing of the yeast *Lachancea lanzarotensis* CBS 12615^T^. The assembly comprises 24 scaffolds, for a total size of 11.46 Mbp. The annotation revealed 5,058 putative protein-coding genes. Detection of seven centromeres supports a chromosome fusion, which occurred after divergence from *Lachancea thermotolerans* and *Lachancea kluyveri.*

## GENOME ANNOUNCEMENT

*Lachancea lanzarotensis* is a newly described species isolated from grapes and wine fermentation during a study of yeast communities in vineyards and wineries in the Canary Islands (1). This species, naturally present in grape must, contributes to spontaneous alcoholic fermentation during the early phases of wine fermentation, before *Saccharomyces cerevisiae* becomes dominant and completes the process. Knowledge of its gene repertoire, especially concerning enzymes involved in fermentation process, will provide clues to understand the wine microbial ecosystem and its functioning, as well as enhance aroma and wine quality.

The CBS 12615^T^ genome was sequenced using Illumina-Solexa technology, from a 500-bp library, on paired-end reads. An ~70-fold coverage was generated. Reads were assembled using SOAPdenovo2 v2.04 (2) with a k-mer size of 75, as recommended by KmerGenie v1.6741 (3). Gaps were filled in using GapCloser v1.12 (2). The rDNA unit was assembled through iterative runs of Newbler v2.7 (http://454.com/products/analysis-software/index.asp) and manually integrated between two scaffolds harboring a partial rDNA unit at one of their extremities. The MAT locus was localized by synteny with that of *Lachancea thermotolerans* (4) and represented as a stretch of five “N” in scaffold 11. Based on the reference genomes of the closely related species *L. thermotolerans* and *Lachancea kluyveri* (4), putative protein-coding genes were annotated using the Amadea annotation transfer tool (Isoft, France). Transposable elements were identified by BLAST with known *Ty1*, *Ty3*, and *hAT* sequences from the *Lachancea* clade as queries (4, 5). tRNA genes were identified using tRNAscan-SE v1.3.1 (6). Additionally, snRNAs were identified by sequence homology with *L. thermotolerans* (4) snRNA sequences. Centromeres were localized by searching for characteristic motifs in syntenic regions of *L. thermotolerans* centromeres (4) using MEME v4.9.1 (7).

The current draft comprises 24 scaffolds interrupted by 52 gaps, for a total size of 11,461,889 bp and a G+C content of around 44.3%. A total of 5,058 putative protein-coding genes have been identified, 321 of which harbor spliceosomal introns within the coding sequences. A total of 59 additional genes have been annotated as dubious models or pseudogenes, with frameshifts, stops in translation, or dubious starts or stops. The genome contains 182 tRNAs. Whenever possible, functional annotation was transferred from *S. cerevisiae* or refseq protein sequences, or experimentally validated proteins from other *Lachancea* species. During this process, 3,863 proteins showed at least 50% sequence similarity with *S. cerevisiae*. Finally, only 48 putative proteins have no known homologs. Class I and class II transposable elements were detected: LTR-retrotransposons with five *Ty3*-like and two *Ty1*-like degenerate copies, as well as 25 copies of *hAT* elements including one putative autonomous copy. Only seven centromeres have been identified, suggesting that *L. lanzarotensis* has one less chromosome than *L. thermotolerans* and *L. kluyveri*. Dot-plot comparison showed that no large DNA deletion occurred, which is compatible with the fact that CBS 12615 has undergone a chromosomal fusion.

Further comparison of the genome of CBS 12615 against other *Lachancea* species will provide additional insights concerning structural genome evolution and its functional impact.

### Nucleotide sequence accession numbers. 

This whole-genome shotgun project (PRJEB7950) has been deposited at the European Nucleotide Archive under the accession numbers LN736360 to LN736383.
